# A Comparison of Adaptive Functioning Between Children With Duplication 7 Syndrome and Williams-Beuren Syndrome: A Pilot Investigation

**DOI:** 10.3389/fpsyt.2022.863909

**Published:** 2022-05-06

**Authors:** Paolo Alfieri, Francesco Scibelli, Federica Alice Maria Montanaro, Cristina Caciolo, Paola Bergonzini, Maria Lisa Dentici, Stefano Vicari

**Affiliations:** ^1^Child and Adolescent Neuropsychiatry Unit, Department of Neuroscience, Bambino Gesù Children’s Hospital, IRCCS, Rome, Italy; ^2^Medical Genetics Unit, Bambino Gesù Children’s Hospital, IRCCS, Rome, Italy; ^3^Genetics and Rare Diseases Research Division, Bambino Gesù Children’s Hospital, IRCCS, Rome, Italy; ^4^Department of Life Sciences and Public Health, Università Cattolica del Sacro Cuore, Rome, Italy; ^5^Centro di Riabilitazione Casa San Giuseppe, Opera Don Guanella, Rome, Italy

**Keywords:** behavioral phenotype, language impairment, adaptive functioning, cognitive functioning, rare genetic syndrome

## Abstract

Interstitial deletions of 7q11.23 cause the well-known Williams–Beuren Syndrome (WBS), while duplication of the same region leads to duplication 7 syndrome (Dup7). Children with WBS share a distinct neurobehavioral phenotype including mild to severe intellectual disability, severely impaired visual spatial abilities, relatively preserved verbal expressive skills, anxiety problems, enhanced social motivation (i.e., hypersociable behaviors) and socio-communicative problems. Children with Dup7 syndrome exhibit some “inverted” features when compared to those of individuals with WBS, such as reduced social motivation and impairment of expressive language. Direct comparison of WBS and Dup7 represents a unique opportunity for the neurobehavioral characterization of the 7q11.23 section. However, most of the available data come from qualitative analysis between different studies. To the best of our knowledge, there are no studies directly comparing features of two matched samples of individuals with WBS and Dup7 syndromes. In this pilot study, we compare the adaptive functioning – measured with the Vineland Adaptive Behavior Scales, Second Edition – of two relatively small samples of children with molecularly confirmed diagnosis of WBS and Dup7 matched for IQ and chronological age, with a particular attention to socialization domain and expressive subdomain. Contrary to our assumption, we have not found any significant difference on socialization domain and expressive subdomains. This pilot investigation suggests that, when matched for chronological age and cognitive level, children with WBS and Dup7 share more similarities than expected. The inverted features that emerge in clinical settings on expressive language and social motivation seem not to differently interfere with the daily abilities to communicate and socialize with meaningful others during daily lives. Differences highlighted by previous undirected comparisons could be due to general and non-specific factors such as cognitive level, which is more severely impaired in individuals with WBS than Dup7. Implications for assessment and treatment are discussed.

## Introduction

Interstitial deletions of 7q11.23 cause the well-known Williams–Beuren Syndrome (WBS) (MIM 194050), while micro-duplication of the same region leads to 7q11.23 duplication syndrome (Dup7). WBS was first described in 1961 by J.C.P. Williams (1) and since the moment in which this non-allelic homologous recombination was reported, it has been supposed that a syndrome with the opposite picture would have existed, even though there were still no information about the phenotype. The first case of Dup7 was described only in 2005, when Somerville and colleagues tested a patient who showed severe expressive speech delay – a feature that was opposite to the linguistic characteristics seen in WBS (2).

In principle, microdeletions and micro-duplications of this specific region on the long arm of chromosome 7 were supposed to occur at the same frequency. However, while the prevalence of WBS is 1 in 7.500 live births, the prevalence of Dup7 is still unknown. It has been proposed that *de novo* prevalence of Dup7 could be 1/12.000, while parental transmission prevalence 1/7.500 live births (3).

The comparison between WBS and Dup7 phenotypes may represent a unique opportunity to better understand the brain functions in which the 7q11.23 section is involved.

Suggestive findings of WBS can be heterogeneous, however, they generally include characteristic “elfin-like” facial features (i.e., periorbital fullness, a stellate/lacy iris pattern, large ear lobes, wide mouth, prominent lips), feeding difficulties in childhood and cardiovascular diseases (usually supravalvar aortic stenosis) and ocular, endocrine and gastrointestinal abnormalities (4). Furthermore, people with WBS share a distinct neuropsychological, cognitive and behavioral phenotype, which counts mild to severe intellectual disability (ID), developmental delay (5), severely impaired visual spatial abilities (i.e., preferential processing of local forms and details; deficits in face processing) (6), anxiety problems, enhanced social motivation (i.e., “hypersociable behaviors,” lack of “stranger danger”) (7), and socio-communicative problems (deficits in shared attention, the understanding of social relationships, etc.) (8). In addition, people with WBS exhibit a characteristic linguistic profile, with receptive skills usually more impaired than expressive ones (9). On the other side, even though language skills have always been considered a strength in the WBS profile, they fail during everyday life-communication and community living, as studies on adaptive functioning showed (10, 11), in particular in adolescence (11).

Children with the reciprocal microduplication do not share distinct physical features, even if some recurring characteristics have been reported, including macrocephaly, broad prominent forehead, elongated palpebral fissures, short philtrum, thin lip vermilion and microstomia (12, 13). Furthermore, children with Dup7 may exhibit developmental delay and ID (usually in the mild range), behavior problems (selective mutism, anxiety disorders), ADHD (Attention Deficit Hyperactivity Disorder) and extreme shyness around strangers (14); moreover, individuals with Dup7 demonstrate language delay, with wide variability ranging from mild to severe expressive impairment (2). The receptive abilities seem to be considerably better than the expressive vocabulary (15).

The comparison of WBS and Dup7 represents an outstanding possibility for the neurobehavioral characterization of the 7q11.23 section. For instance, some “common,” “mitigated,” and “inverted” features have already been revealed. Common features between the two syndromes include anxiety problems and the presence of autistic features. Mitigated characteristics count ID and adaptive impairment, usually mild in children with Dup7 when compared to the ones with WBS. Instead, social motivation and verbal skills are usually considered as inverted characteristics (16, 17). In fact, while children with WBS are described as hyperverbal, hyper-sociable and with relatively spared expressive skills, children with Dup7 usually show selective mutism, social anxiety and impairment in expressive skills. Visuospatial cognition skills seem to be inverted too (spared in patients with Dup7 and impaired in WBS patients) (17).

In order to provide a more realistic clinical picture and then a logical basis for individualized supports, over the last two decades assessment of cognitive functioning has been integrated with measurement of adaptive functioning, which is more representative of behavior during everyday life. Indeed, AAIDD (American Association on Intellectual and Developmental Disabilities) postulates that “*ID is characterized by significant limitations both in intellectual functioning and in adaptive behavior as expressed in conceptual, social, and practical adaptive skills. This disability originates before age 18*” (18). Therefore, adaptive behaviors should be always evaluated during ID assessment.

To our knowledge there are no studies comparing adaptive functioning of the above mentioned rare genetic conditions, therefore the main aim of this cross-syndrome study is to compare the adaptive functioning of two relatively small samples of children with molecularly confirmed diagnosis of WBS and Dup7, matched for chronological age and IQ.

The comparison between adaptive profiles in these syndromes could allow having further information on “common,” “mitigated,” and “inverted” features as expressed in daily life and to better understand if the reciprocal duplication of the region deleted in WBS leads to a different adaptive functioning or if on the contrary the two syndromes have a comparable phenotype. Based on previous studies, we expect to find significant differences in socialization domain and expressive subdomains, where children with WBS should reach better scores.

## Materials and Methods

### Participants

All participants have been recruited at the Child and Adolescent Psychiatry Unit of Bambino Gesù Children’s Hospital. Specifically, our sample includes seventeen participants, nine with molecularly confirmed diagnosis of WBS and eight with molecularly confirmed diagnosis of Dup7 (Table 1).

**TABLE 1:** Cytogenetic and molecular characterization of our cohort of patients affected by 7q11.23 microduplication syndrome and WBS syndrome.

N	Gender	Syndrome	CGH array (start and end point) or FISH analysis	Length of duplicated region	Inheritance
1	M	Dup7	7q11.23 (72,726,578–74,339,044)×3	1,6 Mb	n.a.
2	M	Dup7	7q11.23 (72,726,578–74,339,044)×3	1,6 Mb	paternal
3	M	Dup7	7q11.23 (72,726,578–74,139,390)×3	1,4 Mb	maternal
4	M	Dup7	7q11.23 (72,726,578–74,119,570)×3	1,4 Mb	*de novo*
5	F	Dup7	7q11.23 (72,726,578–74,139,390)×3	1,4 Mb	n.a.
6	M	Dup7	7q11.22q11.23 (72,044,007–74,139,390)×3	2,1 Mb	*de novo*
7	F	Dup7	7q11.23 (72,283,565–74,134,911)×3	1,9 Mb	*de novo*
8	M	Dup7	7q11.23 (72,726,578–74,119,570)×3	1,4 Mb	paternal
9	M	WBS	FISH 7q11.23 deletion	FISH	*de novo*
10	M	WBS	FISH 7q11.23 deletion	FISH	*de novo*
11	M	WBS	FISH 7q11.23 deletion	WBS critical region deleted	*de novo*
12	F	WBS	FISH 7q11.23 deletion	WBS critical region deleted	*de novo*
13	F	WBS	FISH 7q11.23 deletion	WBS critical region deleted	*de novo*
14	M	WBS	FISH and array-CGH negative	frameshift mutation in *ELN* gene (c.205delG, p.Ala71ArgfsTer51)	*de novo*
15	M	WBS	FISH 7q11.23 deletion	WBS critical region deleted	*de novo*
16	F	WBS	FISH 7q11.23 deletion	WBS critical region deleted	*de novo*
17	M	WBS	FISH 7q11.23 deletion	WBS critical region deleted	*de novo*

*N, number; M, male; F, female; Dup7, 7q11.23 Microduplication Syndrome; WBS, Williams Beuren Syndrome; Mb, megabases; n.a., not available.*

Age (Mean [M]; Standard Deviation [SD], MED [Median]) of WBS group was 102.88 (±30.48, MED = 102.88, age range 51–148) months, while Dup7 group was 95.63 (±23.62, MED = 91.5, age range 66–135) months. Cognitive level of WBS group was 70.88 (±14.39, MED = 75, IQ range 49–87), while Dup7 group was 70.37 (±12.80, MED = 70.5, IQ range 47–85). Groups match for chronological age and IQ (*P* values always > 0.05).

### Materials

Adaptive functioning was measured by means of VABS-II (19), a standardized tool developed to measure adaptive behavior and to support diagnosis of ID. VABS-II are widely used in clinical, educational, and research settings and are often considered the “gold standard” instrument for quantifying impairments in adaptive behaviors [i.e., see (20)]. The scale can be administered from birth to 99 years of age and has already been used in several populations, such as ASD [i.e., (21)], Fragile X Syndrome [i.e., (22)] WBS (23–25) as well as Dup7 (13). VABS-II is a semi-structured interview with the primary caregiver evaluating four domains: Communication, Daily Living Skills, Socialization and Motor Skills. Each domain is composed by specific subdomains: Communication (Expressive; Receptive; Written); Daily Living Skills (Personal; Domestic; Community); Socialization (Interpersonal; Play and Leisure; Coping Skills); Motor Skills (Fine Motor; Gross Motor). As Motor Skills domain is usually administered only to children younger than 6 years of age, it has not been included in the statistical analysis of this research.

Obviously, since the expression of adaptive behavior changes across lifespan, in the VABS-II every composite score is age-normalized. This instrument allows to calculating an overall composite score as well as domain- and subdomain-level constructs.

Cognitive level was measured by means of appropriate developmental tools. More specifically, Wechsler scales (WIPPSI-III and WISC-IV) were used with verbally fluent and behaviorally compliant children, while Leiter International Performance Scale was used with children with more severe speech impairment and behavioral difficulties. Given to the extend time of administration, we used two different editions of the Leiter assessment system (Leiter-R and Leiter 3).

WIPPSI-III (26) is an intelligence test designed for children between 2 years and 6 months and 7 years and 3 months age. It provides both Verbal and Non-Verbal IQ, as well as a Full Scale IQ, which is representative of general intellectual functioning.

WISC-IV (27) is a measure of intellectual performance of subjects aged between 6 years to 16 years and 11 months. It allows to calculating four main Reasoning Indices (Verbal Comprehension, Perceptual Reasoning, Working Memory, and Processing Speed) and a Full Scale IQ.

Leiter-R (28) and Leiter-3 (29) are non-verbal intelligence scales, widely used with people with expressive difficulties. In both versions neither the examiner nor the patient are allowed to speak. Leiter-R can be administered from 2 years and 0 months to 20 years and 11 months, while Leiter-3 covers an age range from 3 years to 75 + years.

### Procedure

Current research is a cross-syndrome comparison study of adaptive profiles in children and adolescents with WBS and Dup7 matched for chronological age and developmental/cognitive level. Subjects included in this study were evaluated in Child and Adolescence Psychiatry Unit of Bambino Gesù Children Hospital from 2017 to 2020. Tests were administered during routine clinical activities, with assessment procedures usually lasting 3 working days. Assessment of cognitive and adaptive functioning of Dup7 group was part of a previous larger neurobehavioral investigation on clinical features of children with Dup7 (13). Thus, we selected 9 children with WBS from a database including 63 children evaluated from 2012 to 2020.

We chose those patients that received a full evaluation of cognitive and adaptive functioning and that could have been matched with the ones with Dup7 for age and IQ.

Since Wechsler scales provide a general estimate of intelligence, while Leiter assessment system measures only non-verbal intelligence, children from the two groups were matched also for the cognitive test that was used for the evaluation. In two cases, since the same assessment systems were not available, we used the Visual Spatial Index of WISC-IV and the Performance IQ of WPPSI-III, which provide an estimate of non-verbal intelligence and we matched it with the IQ provided by Leiter-3. Cognitive tests were distributed as follow: WBS (2 WISC-IV, 1 WPPSI-III, 4 Leiter 3, 2 Leiter-R), Dup7 (3 WISC, 5 Leiter 3).

Assessment was conducted by a team of trained and specialized child psychiatrists and psychologists and consisted of clinical observations, standardized evaluations and parent interviews. All parents signed an informed consent for research purpose.

The study was approved by Ethical Committee of Bambino Gesù Children’s Hospital (number of protocol: 1125).

### Statistical Analyses

Descriptive statistics (MED, M, min-max; SD) were elaborated for VABS Adaptive Behavior Composite (ABC), domains and subdomains scores. Raw scores of VABS domains and subdomains were converted to standard scores (IQ, ABC and VABS II domains scores have a M of 100, and SD of 15 while subdomains scores have a M of 15 and SD of 3). Normalized scores were used in all analyses.

To analyze differences between WBS and Dup7 groups in the domains and subdomains of adaptive behaviors measured with VABS II, Mann–Whitney *U* test was performed with Group (WBS vs. DUP7) as independent variable and VABS II domains and subdomains scores as dependent variables; then, a between – group design was used. A *P* value ≤ 0.05 was considered as statistically significant.

Furthermore, to detect strengths and weaknesses within subcomponents of VABS II in children with WBS or DUP7, the Friedman Test was conducted, with VABS II domains (Communication, Socialization, and Daily Living Skills) as within-subject factors. Then, Wilcoxon signed-rank tests were used to analyze differences between pairs of VABS II domains. Bonferroni’s correction was applied to the Wilcoxon signed-rank tests in order to correct alpha (alpha corrected = 0.016). After correction, a *P* value < 0.016 was considered statistically significant. All data analyses were performed using STATISTICA Six Sigma, STATISTICA release 7 (StatSoft, Inc., 1984–2006).

## Results

### Adaptive Domains and Subdomains in VABS II – Between Groups’ Comparisons

All descriptive statistics of two groups are presented in Tables 2, 3.

**TABLE 2:** Descriptive statistic and comparison between groups in VABS II Domains.

	Dup7 group				WBS group					
VABS-II Domains	MED	M	Min-Max	SD	MED	M	Min-Max	SD	*z* adjusted	*P* level*
Communication	53.00	53.38	40.00–71.00	10.41	60.00	57.44	41.00–80.00	12.38	0.7	0.47
Daily living skills	65.50	66.25	42.00–107.00	22.30	50.00	51.78	42.00–73.00	10.11	−1.3	0.19
Socialization	69.00	70.75	54.00–87.00	11.72	66.00	69.00	61.00–85.00	7.11	−0.24	0.81
ABC	59.50	59.25	38.00–85.00	14.97	52.00	52.22	41.00–74.00	9.42	−1.05	0.29

ABC, Adaptive Behavior Composite; MED, median; M, media; Min-Max, Minimum-Maximum; SD, standard deviation, *significant at P ≤ 0.05.

**TABLE 3:** Descriptive statistic and comparison between groups in VABS II Subdomains.

	Dup7 group				WBS group					
VABS II Subdomains	MED	M	Min-Max	SD	MED	M	Min-Max	SD	*z* adjusted	*P* level*
Receptive	8.50	8.38	6.00–10.00	1.77	9.00	8.89	7.00–11.00	1.62	0.54	0.58
Expressive	8.00	7.88	4.00–12.00	2.80	9.00	9.45	7.00–13.00	1.81	1.11	0.26
Written	7.00	7.38	2.00–11.00	3.02	9.00	7.11	2.00–14.00	3.92	−0.29	0.76
Personal	4.50	6.63	2.00–13.00	5.13	2.00	3.12	2.00–10.00	2.62	−1.66	0.09
Domestic	11.50	12.75	9.00–20.00	3.73	12.00	11.44	9.00–14.00	1.81	−0.4	0.62
Community	7.50	8.13	4.00–15.00	3.27	5.00	6.22	5.00–8.00	1.48	−1.2	0.19
Interpersonal	8.50	8.63	4.00–14.00	3.50	8.00	8.44	6.00–13.00	1.88	−0.15	0.88
Play and leisure	9.00	9.00	7.00–11.00	1.31	10.00	9.11	7.00–11.00	1.36	0.19	0.84
Coping skills	9.00	9.63	7.00–13.00	2.13	9.00	8.67	7.00–12.00	1.50	−0.84	0.39

MED, median; M, media; Min-Max, Minimum-Maximum; SD, standard deviation, *significant at P ≤ 0.05.

There was no significant group difference on overall adaptive outcomes and on VABS II domains and subdomains (*P* > 0.05). A slight slide toward significance (*P* = 0.26) was revealed in Communication – Expressive Skills Subdomain, which scores are higher in WBS group (MED = 9, *M* = 9.45) than in Dup7 one (MED = 8, *M* = 7.88). Another approaching but not reaching significance result was observed in Daily Life Skills – Community subdomain (*P* = 0.19), where Dup7 (MED = 7.15, *M* = 8.13) perform better than WBS people (MED = 5, *M* = 6.22). Finally, a statistical trend toward significance was found in Daily Living Skills – Personal Subdomain (*P* = 0.09), with WBS group (MED = 2, *M* = 3.12) performing worse than Dup7 one (MED = 4.5, *M* = 6.63).

### Adaptive Domains in VABS II – Within Groups’ Comparisons

**FIGURE 1:**
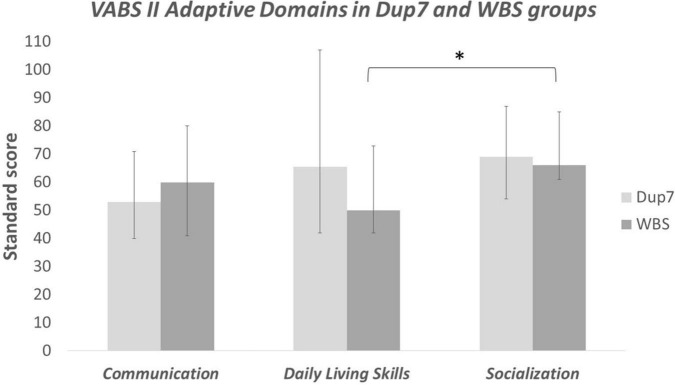
Adaptive Domains in VABS II – within groups’ comparisons; *significant at *P* ≤ 0.05.

Considering Dup7 group, no significant differences were found between Communication, Socialization and Daily Life Skills Domain in any of the three comparisons. *P* value always > 0.05 (see Figure 1).

Taking into account WBS group, no significant differences emerged between Communication and Daily Life Skills. On the other side, results of Wilcoxon signed-rank test revealed significant differences between Communication and Socialization Domains (*P* = 0.045); however, significance was no longer present after Bonferroni’s adjustment (*P* 0.045 > 0.016). A significant difference emerged between Daily Life Skills and Socialization Domains (*P* = 0.007), that persisted also after Bonferroni’s correction (*P* 0.007 > 0.016).

## Discussion

Despite on the greater or lesser knowledge on adaptive functioning on WBS and Dup7 alone, no cross-syndrome comparisons examining adaptive behavior have been carried out. However, delineating adaptive trends and potential phenotypic specificity between rare genetic syndromes is crucial to improve clinical-functional diagnosis and develop novel early interventions.

This brief report is the first attempt to investigate differences on adaptive functioning in two small samples of children with deletions of 7q11.23 and with microduplications of the same region.

Results showed that non-significant difference emerged between the two groups in domains, subdomains and in Adaptive Behavior Composite. These results are somehow unexpected, given the differences highlighted in the previous studies on neuropsychological and psychopathological features of the two syndromes [i.e., (15)]. On the other side, it has to be taken into account that the absence of significant results could be due to the small sample size; effectively, we selected a group of nine WBS to compare with a group of eight Dup7. Since these syndromes are particularly rare and considering the methodological decision to match individuals not only for age, but also for IQ, our sample size was inevitably small. This specification has to be considered when interpreting results.

Our findings could suggest that differences between these conditions could be due to other features such as cognitive level rather than to the genotype. Previous observation reporting that children with WBS usually have a worsening functioning in adaptive skills when compared to children with Dup7 could depend on the widest presence of severely cognitive impairment in population of individuals with WBS (14). However, when children with WBS are matched with Dup7 for chronological age and IQ, these differences in adaptive level seems to disappear.

Analysis of domains and subdomains also revealed interesting information. The absence of difference in Socialization was not expected. The inverted hyper-sociable (WBS) versus inhibited/social anxious behaviors (Dup7) profile, which has been reported in previous studies, in our groups seems not to conduct to different patterns in establishing relationships with peers and adults.

Furthermore, even though in our sample there is not a significant difference in Communication domain and subdomains between WBS and Dup7, expressive subscale of WBS seems to be more preserved than the one of Dup7, consistently with literature considering expressive skills of children with WBS and Dup7 as opposite (relatively preserved in WBS and severely compromised in Dup7). However, reminding that both populations exhibit a performance below 2 SD in each subdomain of Communication, a possible interpretation could be that children with Dup7 are not so more impaired in expressive skills than the ones with WBS as usually thought. For this reason, assessment of speech and language characteristics should include also other information; in fact, high level of social anxiety and selective mutism, as well as Disruptive Behavior Disorders (typical of children with Dup7), could at least partially account for this discrepancy. Moreover, while children with WBS are generally compliant with the examiner, children with Dup7 may show oppositional or inhibited behaviors that could lead to a poorer performance in structured evaluations. Generalized anxiety could contribute to the worsening of performance on language tests as well.

On the other side of the coin, children with WBS, which expressive abilities seem to be less impaired, when are evaluated with structured scales, show their real difficulties. For instance, they perform worse than people with Dup7 on tests that measure pragmatic and receptive skills that in WBS are usually more impaired than in Dup7. However, since VABS II do not deeply investigate pragmatic issues, potential significant differences may have not been emerged.

One previous study documented that communication skills measured by VABS II decrease during adolescence period in concomitance with enhancement of social demands, because of a specific worsening of receptive subdomain probably related to pragmatic skills (11). Due to the different language profiles, we would have expected differences between our two groups in the subdomains of communication but this did not happen. However, it should be noted that in our two samples the average age was about 10 years, so it cannot be excluded that the difference was not found due to the young age of our patients.

These considerations could suggest that assessment of language skills should always include batteries that measure pragmatic and daily-life usage of language. Likewise, evaluations should be conducted in a wider period in order to allow children with Dup7 to become more familiar with the clinical setting. In fact, only a more comprehensive evaluation could be able to clarify the reasons of eventual differences in language between those populations.

Furthermore, a slightly trend toward significance was found in Daily Life Skills – Personal Subdomain, which may indicate that WBS people probably show more difficulties in eating, dressing, self-care and personal hygiene than individuals with Dup7. Another difference, even though not achieving acceptable levels of statistical significance, was observed in Daily Life Skills – Community Subdomain, which may imply that WBS perform worse than Dup7 group in the ability to use money, to order food, to use technology and to read a clock. These tiny differences could be due to the visuo-spatial and coordination deficits largely described in WBS population [i.e., (30)].

Concerning within groups’ comparisons, analysis of VABS-II domains did not show significant differences in Dup7 group. Previous studies underlined that Dup7 profile is characterized by a weakness in language abilities, in particular in expressive skills (15). Our results, even though failing to reach statistical significance, are consistent with this finding, in fact in Dup7 group Communication Domain is the most impaired than other domains (Communication MED = 53, *M* = 53.38; Socialization MED = 69, *M* = 70.75; Daily Living Skills MED = 65.5, *M* = 66.25).

Taking into account WBS population, research has demonstrated that WBS is associated with deficits in adaptive functioning (31), where adaptive profile of children with WBS seems to be characterized by relative strengths in the Socialization and Communication domains and challenges in Daily Living Skills and Motor functioning (32). Our study is consistent with this line of evidence, in fact performance on Socialization Domain was significantly higher than Daily Living Skills one. This may indicate that the hyper-sociable behavior and the apparent expressive skills of WBS people may hide their real difficulties in adaptive functioning.

In conclusion, this pilot investigation suggests that, when matched for chronological and cognitive level, children with WBS and Dup7 share more similarities than thought. Differences that usually emerge in the clinical evaluation of expressive language and social motivation seem not to come up in the daily abilities to communicate and socialize with meaningful others. Then, differences popped out in previous indirect comparisons could be due to general and non-specific factors such as cognitive level, which is more severely impaired and inhomogeneous in individuals with WBS than in the ones with Dup7, rather than on syndrome-specific features. Therefore, this evidence could suggest that interventions for patients with WBS and Dup7 should target the same adaptive skills across all domains.

Our study has some limitations. First, the relatively small sample size may have not allowed to detect differences present in the general populations. Future studies on wider groups are then required to better compare the adaptive profiles of children with WBS and Dup7. Second, since adaptive behavior may change on the basis of different factors such as early interventions, it could be interesting to evaluate if other aspects influence the performance on VABS-II. Third, as we used developmentally appropriate cognitive assessment, different tests have been used to measure cognitive/developmental levels (i.e., Wechsler Scales and Leiter-3) that may have led to a heterogeneous evaluation. Then, future cross-syndrome studies should include a larger sample size and matched patients for the greatest number of features possible.

To conclude, using a cross-syndrome comparison approach revealed partially overlapping profiles in WBS and Dup7, despite the two groups being considerate opposite syndromes. However, as a whole, the two disorders remain distinct in the severity of their core difficulties. This study has some limitations, given the relatively small sample of evaluated subjects. Future studies on larger number of individuals with 7q11.23 deletion and duplication are necessary to support our data and deeper analysis is required to better investigate their adaptive functioning and to design innovative specific diagnostic measures and early novel interventions.

## Data Availability Statement

The raw data supporting the conclusions of this article will be made available by the authors, without undue reservation.

## Ethics Statement

The studies involving human participants were reviewed and approved by the Ethics Committee of the Bambino Gesù Children’s Hospital number of protocol 1125. Written informed consent to participate in this study was provided by the participants’ legal guardian/next of kin.

## Author Contributions

PA and FS conceived the study. PA, FS, and FAMM wrote the manuscript. CC and FAMM analyzed the data and assisted in the interpretation of findings. SV, PA, PB, and MLD critically reviewed the manuscript. All authors are agreed to be accountable for all aspects of the work in ensuring that questions related to the accuracy or integrity of any part of the work are appropriately investigated and resolved, they provide intellectual input and approved the final manuscript.

## Conflict of Interest

The authors declare that the research was conducted in the absence of any commercial or financial relationships that could be construed as a potential conflict of interest.

## Publisher’s Note

All claims expressed in this article are solely those of the authors and do not necessarily represent those of their affiliated organizations, or those of the publisher, the editors and the reviewers. Any product that may be evaluated in this article, or claim that may be made by its manufacturer, is not guaranteed or endorsed by the publisher.
